# What Makes Canine Search and Rescue Successful? Insights into Environmental, Management, and Personality Factors

**DOI:** 10.3390/ani16040664

**Published:** 2026-02-19

**Authors:** Silvia Silvestri, Gabriele Brecchia, Olimpia Barbato, Alda Quattrone, Marco Valsecchi, Laura Menchetti

**Affiliations:** 1 School of Biosciences and Veterinary Medicine, University of Camerino, Via Circonvallazione 93/95, 62024 Matelica, Italy; silvia.silvestri25@gmail.com; 2 Department of Veterinary Medicine, University of Perugia, Via San Costanzo 4, 06126 Perugia, Italy; gabriele.brecchia@unipg.it; 3 Department of Veterinary Medicine and Animal Sciences, University of Milan, Via dell’Università 6, 26900 Lodi, Italy; alda.quattrone@unimi.it; 4 Associazione Nazionale Carabinieri, Via Carlo Alberto Dalla Chiesa 1/A, 00192 Rome, Italy; delegatonazionaleucrs@cinofiliassocarabinieri.it

**Keywords:** working dogs, search and rescue dogs, motivation, sociability, sensitive developmental phases, critical periods of development, personality traits, high temperature, handler–dog relationship, performance assessment

## Abstract

This study examined the effects of environmental conditions, behavioral history, management practices, and personality traits on the operational performance of search and rescue (SAR) dogs and dogs admitted to SAR certification testing. Thirty-two handlers completed a questionnaire collecting demographic data, as well as information on their dogs’ behavioral history, management practices, and personality descriptors. Each dog–handler unit also undertook a search trial consisting of locating a hidden person in a wooded area, which was evaluated both by professional instructors and the handlers through ratings of critical behavioral indicators. Objective measurements were obtained through a weather station and GPS devices. Handlers described their dogs mainly in terms of work-relevant traits, such as socio-cognitive engagement, assertiveness, and arousal. The performance evaluation form was practical and efficient, though the *Distraction* parameter may require refinement, and handler ratings suggested a self-reporting bias. Temperature and wind speed were negatively associated with performance, whereas higher humidity was positively associated with it. Performance was also associated with litter size, age at adoption, dog experience, and management-related factors. Finally, speed, ground coverage, and a canine profile characterized by high arousal and reactivity were strong determinants of good search performance (|ρ| ≥ 0.3; *p* < 0.05). Although these findings require confirmation in larger samples, search performance appears to be a multifactorial construct shaped by the interplay of extrinsic and intrinsic factors. Defining the contribution of each factor could help optimize performance and dogs’ welfare.

## Simple Summary

Search and rescue (SAR) dogs play a crucial role in disaster response and missing person searches. Despite their high social value, however, research on the factors influencing SAR dog performance remains limited. We investigate key factors affecting operational performance in search dogs, including working environment, experience, and behavioral profiles. Information on the behavioral history, management, and personality of SAR dogs and dogs admitted to SAR certification testing was collected. The dogs then performed a simulated search for a missing person. Performance was evaluated using behavioral indicators and data collected via GPS devices. In our sample, associations were found between temperature, humidity, wind, and dogs’ behavior, search strategy, and detection ability. The performance of dogs was also shaped by management practices, including early socialization, as well as by personality traits. Understanding the contribution of these factors may support the development of evidence-based guidelines for dog selection and management, ultimately optimizing both performance and welfare. Nevertheless, further studies with larger sample sizes are needed, and greater research efforts should be directed toward this topic.

## 1. Introduction

Search and rescue (SAR) dogs make a valuable contribution during disasters and in locating missing persons thanks to their ability to detect human scent across a wide range of substrates (in air and beneath rubble, snow, ash, mud or water) and environments (natural and anthropogenic) [1]. Their outstanding detection performance is largely attributable to the anatomical and physiological specialization of the canine olfactory system [2,3]. However, these features, which are shared by all macrosmatic species, are not sufficient to explain the operational role of the SAR dog, which, in the face of technological advances, has not yet been replaced by any artificial device.

Despite their long-standing use and recognized social value across various contexts, scientific interest in SAR dogs has emerged only relatively recently [4]. Consequently, many management practices continue to rely primarily on handlers’ empirical knowledge, leaving substantial scope for research to optimize canine performance while preserving dogs’ welfare. The existing literature, comprising both experimental studies [5,6,7,8,9] and perspective articles [10,11,12], suggests that search performance can be influenced by several factors, including extrinsic conditions (e.g., environmental characteristics), handler-related variables (e.g., training approaches, management strategies, handler’s beliefs or disposition), and intrinsic canine attributes such as personality traits and physical fitness. However, investigating this topic presents substantial methodological challenges [13], particularly in designing research protocols that are both standardized and ecologically valid for real-world operational contexts. As a result, most studies tend to examine isolated factors or rely on tasks conducted in controlled environments, despite the fact that search performance in real-world contexts emerges from a complex interaction of multiple factors.

Dogs locate human scent as odor plumes, which represent a complex mixture of volatile organic compounds (VOCs), skin rafts (fragments of dead skin cells), and aerosols. All components of human scent plumes may be chemically altered and differentially transported depending on environmental temperature and humidity, wind, ground conditions, and the characteristics of the search area (e.g., vegetation or building configuration) [2,5,14]. Such environmental factors may therefore facilitate, hinder, or in some instances even preclude accurate odor source localization, thereby impairing overall olfactory search performance. Accordingly, environmental variables should be systematically included in all field-based studies.

The localization of the odor source further depends on the inter- and intra-breed variability in dogs’ sniffing physiology and olfactory system structures, including the size of the olfactory epithelium and the number and diversity of olfactory receptors [2,15]. Although the genetic basis of these anatomical and physiological traits has been demonstrated, comparative studies across breeds have yielded contradictory and inconclusive results [16,17,18]. These inconsistencies likely reflect not only the technical challenges of elucidating genetic and anatomical determinants but also the influence of personality on search performance, which can confound breed-level evaluations and help explain the considerable interindividual variation [19].

The personality traits currently identified as desirable in SAR dogs include sociability, trainability, appropriate levels of energy, predatory and play motivation, problem-solving ability, boldness, and a strong general propensity for olfaction [6,10,11]. Beyond instinctive components, the behavioral repertoire of SAR dogs is further shaped by age, environmental and developmental experiences during sensitive periods, and the specific training methods adopted throughout their formative preparation for operational tasks [19,20,21]. Collectively, these aspects may influence multiple dimensions of search performance, which in turn reflect the core requirements of a SAR dog.

A further methodological challenge is represented by performance assessment methods. Certified SAR dogs, according to the Federal Emergency Management Agency [22], must demonstrate reliable command control, agility, a targeted alert bark to indicate detection, and persistence in searching despite potential distractions. They must also demonstrate sufficient confidence to search independently and to cope with challenging and unpredictable environments. In line with this, Clark, Rooney, and colleagues [23,24,25] have validated a practical assessment tool for evaluating search performance. The tool is based on ratings of behavioral measures observed during search tasks, focusing on dimensions including control, motivation, distractibility, search pattern, stamina, strength of indication, confidence, search accuracy, independence, speed, and the ability to detect and accurately locate a target. The development of feasible, straightforward tools that enable performance assessment independent of the operational context, alongside tools for collecting information on behavioral history, handling practices, and personality, may help clarify the factors influencing performance and support the establishment of evidence-based guidelines for the selection and management of SAR dogs.

We hypothesized that canine performance, assessed through the behavioral indicators critical for search, could be influenced by environmental factors and by the dog’s management and personality. Thus, this study investigated the effects of environmental factors, behavioral history, management practices, and personality traits on dogs’ performance during simulated missing person detection tasks. Furthermore, the validity of the form used to rate critical behavioral indicators of performance was verified using inter-rater reliability between professional instructors and handlers, as well as its associations with GPS-derived movement metrics.

## 2. Materials and Methods

This study was conducted in August 2024 at the training center for search and rescue (SAR) canine units of the Associazione Nazionale Carabinieri (ANC), located in Campodolcino (1071 m a.s.l.), Sondrio Province, Italy. The ANC Canine Unit is a specialized volunteer team composed of trained handlers and their dogs. They operate in various domains, including search and rescue, civil protection, and public safety. These dedicated volunteers support emergency services during natural disasters, assist in locating missing persons, and participate in community events and training exercises [26]. The present research was carried out under a collaborative agreement between the University of Camerino (Italy) and the ANC, as a part of a broader research project entitled *Factors Influencing the Performance of Search and Rescue Dogs: A Multivariate Approach Including Personality, Handler–Dog Relationship, Experiences, and Environment*. The study received approval from the Institutional Bioethics Committee of the University of Camerino (protocol No. 3-2024). All participants provided written informed consent for the processing of personal data.

The experimental protocol consisted of two main components: the administration of a questionnaire to the handlers and a simulated search exercise involving the dog–handler units.

### 2.1. Animals

This study involved 32 dogs from the ANC Canine Units, all of which were participating in training and selection sessions. Among them, 5 were already operational, while 27 were not operational but were admitted to the certification test as SAR dogs. Detailed demographic characteristics of the dogs are reported in the Results section and Table S1. All dogs lived year-round with their handlers and came from different regions of Italy. Dog–handler units arrived at the training center the day before the trial, with travel times ranging from 2 to 18 h. During the study, dogs were housed individually in transport vehicles, in accordance with standard procedures used during training sessions and rescue operations. They were fed their regular commercial dry diet, and the supervising veterinarian verified, through clinical examination, that all dogs were in good health.

### 2.2. Questionnaire Content and Structure

The questionnaire (Figure S1) was designed to evaluate several key aspects of SAR dogs and dogs admitted to SAR certification testing, collecting demographic information of both handlers and dogs, management practices, and personality. It included a combination of open- and closed-ended questions and was organized into the following sections:

- **Demographic Information of Handler and Dog.** This section collected handlers’ demographic data, including gender, age, occupation, length of dog ownership, number of dogs and other animals in the household, and family composition. It also covered home environment (e.g., apartment, house with garden, rural or urban setting) and identified the person primarily responsible for the dog’s daily care. Additional questions addressed the handler’s experience in search and rescue, motivations for engaging in this activity, and satisfaction with the experience.
  Dog-related information included sex, gonadectomy, age, breed, weight, origin (e.g., breeder, private owner, shelter), the reason for acquisition, and current and past health status.
- **Behavioral History: Early-Life Experiences and Current Management.** This section was adapted from the “Canine behavioral history form” presented by Martin and Shaw [27] and by previous questionnaires [28,29]. This section collected information on environmental factors and experiences during the dog’s sensitive developmental periods, as well as information about the dog’s current management. Key questions included age at separation from the litter, age at adoption, litter size, whether the puppy was raised with the mother and siblings, previous ownership or rehoming, and any suspicion of prior mistreatment. The current living environment was assessed through questions on sleeping arrangements, daily routine, feeding practices, and time spent alone.
  Two additional sub-sections captured physical activity (type and frequency) and search training (frequency, number of trainers, training level, age at training onset, and concurrent activities). Handlers also indicated which aspects of activity and training their dog seemed to enjoy most.
- **Assessment of Temperament and Behavior.** This section evaluated the dogs’ temperament and personality traits. In the first part, handlers described their dog using three adjectives in free text. The second part consisted of a list of 33 descriptors, from previous canine personality studies [28,30], which handlers rated on a 6-point scale ranging from 0 (strongly disagree) to 5 (strongly agree).

### 2.3. Search Trial

Each dog–handler unit participated in a search trial, conducted blind for both the dog and the handler. The trial was designed to replicate operational search conditions and was therefore carried out using standard search equipment. Handlers wore a backpack and protective helmet and carried an emergency kit containing a radio, GPS device, first-aid kit, and harness, while dogs were equipped with a harness and a signaling device.

**Figure 1:**
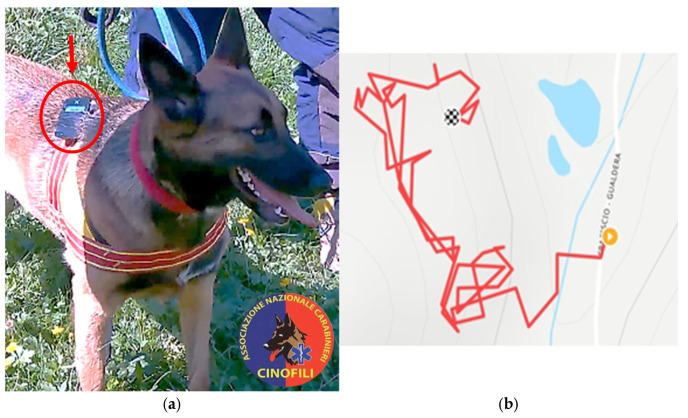
Placement of the GPS device on the dog’s harness (**a**) and example of the GPS track recorded during a search trial (**b**). Photo courtesy of the Associazione Nazionale Carabinieri (ANC).

In addition to the standard equipment, a GPS device (XOSS G + GPS Bike Computer, Hong Kong, China) was secured to the dog’s harness (Figure 1a) to record average speed, maximum speed, moving time, and elevation gain during the search. An example of the track recorded by GPS during the search trial is shown in Figure 1b. To minimize bias due to potential delays in stopping the recording, we computed the ratio between the distance covered and the latency (i.e., the time required for the dog to locate the figurant). This ratio served as a proxy for the effective ground explored by the dog during the active search phase and was defined as the Ground Exploration Index.

**Figure 2:**
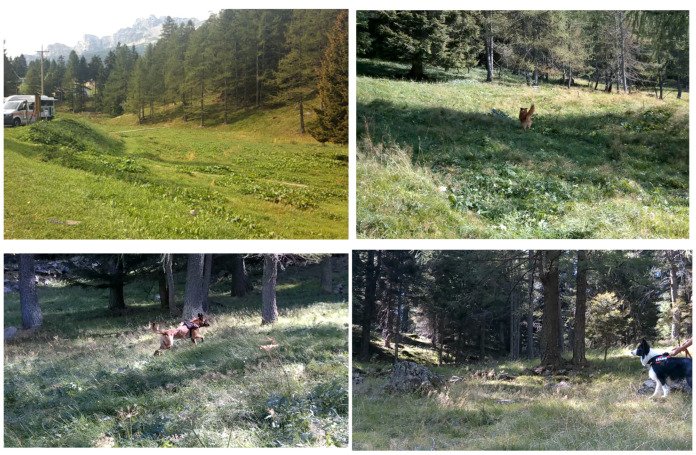
Representative woodland areas used for the search trial. Photo courtesy of the Associazione Nazionale Carabinieri (ANC).

The search trial was conducted in one of four areas selected by two expert instructors, which were predominantly wooded, with limited open-field sections in the initial search area. Different areas were assigned to each dog–handler unit to minimize the risk of handler familiarization with the search environment, as handlers were present during other teams’ trials. All areas were of similar size (approximately 20,000 m^2^ each) and included a small flat section followed by a slope with an inclination of up to 45° (Figure 2).

The objective of the trial was for the dogs to locate a hidden person (figurant) within the assigned field within a maximum time of 20 min. Trials were organized and supervised by senior instructors, who determined the figurant’s location and instructed them on the appropriate behavior once approached by the dog, typically simulating casualty conditions such as unconsciousness or visible distress. The figurant was positioned at an approximate Euclidean distance of 300–500 m from the starting point, so as to remain out of sight. Typical hiding places included recesses among bushes, behind trees or large rocks, inside small caves, or within wooden shelters. Each figurant carried a two-way radio for emergency communication and an emergency kit and was instructed to remain silent.

Before the search, each handler participated in a short briefing with the search operation coordinator (i.e., instructor), mirroring procedures used in real search scenarios. During this briefing, the handler requested key information in accordance with standard protocols, including details about the missing person (e.g., physical characteristics, mobility), the circumstances of the disappearance (e.g., time since last seen), and the boundaries of the search area. During this phase, the handler kept the dog on a leash; once the briefing concluded, the dog was released to conduct the search off-leash. One instructor followed the dog–handler unit at a distance without interfering, while the other remained at the starting point, maintaining radio contact with all participants to receive any reports of difficulties from either the search unit or the figurant.

When the dog–handler unit located the figurant within the established search time frame, the trial was classified as a ‘Success’. Latency was measured from the start of the search task (i.e., the moment the handler released the dog from the leash) until the handler communicated the correct location of the hidden person via radio, using GPS coordinates. Regardless of whether the trial was completed within the time limit, handlers were permitted to continue searching until the figurant was found and to reward their dogs, thereby ensuring a positive trial conclusion.

A weather station (Levenhuk Wezzer PRO LP330, Levenhuk, Inc., Tampa, FL, USA) was placed within the search area, near the starting point, to monitor environmental parameters without providing any cues to either the handler or the dog. Data were recorded at 5 min intervals, downloaded in Excel format, and the environmental parameters corresponding to the search trial were extracted for analysis. During the trials, temperature ranged from 14 to 24 °C, humidity from 59% to 85%, wind speed from 0 to 15 km/h, and atmospheric pressure from 867 to 870 hPa (Table S2).

### 2.4. SAR Dog Performance Evaluation Through Behavioral Parameters

**Figure 3:**
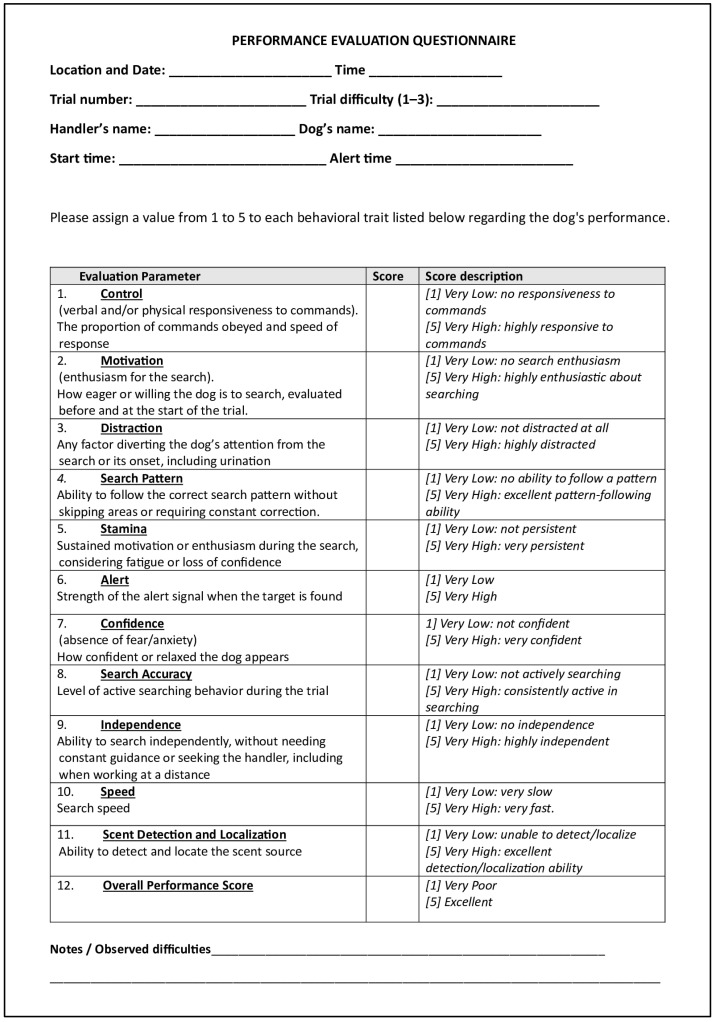
Performance evaluation form used by senior instructors and handlers to assess the dog’s behavior during the search trial. The form includes 11 behavioral parameters and one overall performance score, each rated on a 5-point scale. The right-hand column provided an italicized description of the scoring criteria for each item. Consensus scores were recorded by the senior instructors, while handlers completed the form independently and were blinded to the instructors’ evaluations. The form also included a section to report trial difficulty and any observed issues.

Dog performance was evaluated by two senior instructors, one military and one civilian, each with over 10 years of experience in training SAR dog handlers. Assessments followed the framework developed and validated by Clark and Rooney [23,24,25]. Before the trials, senior instructors and researchers participated in a briefing to ensure consistent interpretation of all behavioral parameters, which included *Control, Motivation, Distraction, Search Pattern, Stamina, Indication, Confidence, Search Accuracy, Independence, Speed, and Ability to Detect and Locate*. The scoring system was based on the assignment of a score ranging from 1 to 5 (1 = very low; 5 = very high) for each parameter (Figure 3).

After each search trial, the two highly experienced instructors initially scored the parameters independently and subsequently shared their evaluations. Discrepancies between raters were infrequent and, when present, were limited to a one-point difference. These differences were easily resolved through a brief discussion focused on the strengths and weaknesses displayed by the dog with respect to the specific items being evaluated. This process consistently resulted in a single consensus score for each parameter, which was used in all subsequent analyses. The consensus scores were recorded on the evaluation form (Figure 3), which included parameter definitions and a section for trial difficulty, accounting for differences in terrain, distance, and figurant concealment. These contextual factors were considered to promote standardization and reduce variability across trials.

Due to differences in figurant placement and terrain, latency was not included as a performance parameter. However, it was indirectly accounted for in the performance evaluation, primarily through the *Scent detection and localization* parameter, which most directly reflected whether the dog successfully located the figurant, and secondarily through *Search accuracy* and *Speed*. In addition, failure to localize the figurant within 20 min was indirectly reflected in the GPS-derived *Ground Exploration Index*, as unsuccessful searches were characterized by a limited explored area relative to the time spent searching.

Handlers completed the same evaluation form immediately after their trial, providing independent assessments of their dog’s performance across the same parameters.

### 2.5. Statistical Analysis

The questionnaire results were initially expressed as mean, minimum and maximum values, frequencies, and percentages. Certain variables, such as age and breed, were further categorized to facilitate subsequent inferential analyses. In particular, to ensure adequate sample sizes within each category, breeds were grouped based on functional origin (e.g., herding, retrieving, utility/defence) and morphological similarities into the following groups: Border Collies and Australian Shepherds; Retrievers (Labrador and Golden Retrievers); German Shepherds (including Sable German Shepherd) and Belgian Malinois; Mixed-breeds; Other.

The adjectives elicited from handlers to describe their dogs’ personalities were reported as absolute frequencies and ordered from the most to the least frequently mentioned. No further standardization procedures were applied. These adjectives were additionally used to generate a word cloud using an online tool (https://wordart.com/create; accessed on 2 December 2025), in which font size was proportional to the frequency of each adjective.

Results of the proposed 33 descriptors rated on a Likert scale were first presented as median, first (Q1), and third quartiles (Q3). Then, a hierarchical cluster analysis (HCA) was performed to identify groups of dogs with similar behavioral profiles. To enable clustering of variables, a proximity matrix was first computed, specifying squared Euclidean distance. Subsequently, a hierarchical cluster analysis was performed using the Ward’s method of linkage. A dendrogram was generated to visually explore how the descriptors grouped together. This procedure enabled the identification of clusters of descriptors with similar scoring trends, providing insights into underlying dimensions of behavior captured by the rating scale. In particular, the dendrogram generated by the analysis was cut at a rescaled linkage distance preceding a marked increase in fusion coefficients (i.e., ≥2 on the rescaled distance axis), yielding a five-cluster solution. For each cluster, a composite score (i.e., cluster score) was computed for every dog by calculating the mean of the variables included in the cluster [31].

The performance scores rated by the instructors were first compared across the different behavioral parameters using a Friedman test. This test evaluates whether there are statistically significant differences in the distributions of scores across the different performance dimensions assessed on the same dogs.

The agreement between the performance scores provided by the instructor and the handler (i.e., inter-rater reliability) was assessed using Krippendorff’s alpha (Kα), with 1000 bootstrap samples to estimate the 95% confidence interval. The Kα coefficient was interpreted as follows: none to slight (0.01 ≤ Kα ≤ 0.20), fair (0.21 ≤ Kα ≤ 0.40), moderate (0.41 ≤ Kα ≤ 0.60), substantial (0.61 ≤ Kα ≤ 0.80), and almost perfect (Kα ≥ 0.81) agreement [32,33,34].

Finally, cluster and performance scores were analyzed to assess their associations with various handler- and dog-related characteristics. Sex and gonadectomy status were evaluated using independent-samples *t*-tests, whereas the effects of age, breed group, time since the start of search training (categorized), and daily walk duration (categorized) were assessed using one-way ANOVA. Spearman’s rank correlation coefficient (ρ) was used to examine associations with continuous and ordinal variables, including the handler’s age, number of dogs and cats in the household, years of handling experience, time since the dogs start of search training, number of children in the household, and the dog’s age at separation from the litter and age at adoption. Spearman’s ρ was also used to evaluate the associations between performance scores and the scores of the behavioral traits extracted through HCA. Correlation strength was interpreted as follows: poor if ρ < 0.3, moderate if 0.3 ≤ ρ < 0.5, and strong if ρ ≥ 0.5 (absolute values considered in all cases) [33,35,36]. Given the large number of correlations, these correlation analyses were exploratory and aimed at identifying potential patterns rather than testing confirmatory hypotheses. Accordingly, individual *p*-values were used to evaluate distinct associations but should be interpreted with caution due to the increased risk of Type I error associated with multiple comparisons. To further account for multiple testing, false discovery rate (FDR) correction using the Benjamini–Hochberg procedure was also applied.

All statistical analyses were performed using IBM SPSS Statistics version 25 (IBM Corp., Armonk, NY, USA). The visualization of the correlation tables as heat maps was performed using OpenAI’s ChatGPT (GPT-5). The AI tool was employed solely for graphical visualization and did not contribute to data analysis or interpretation. A significance threshold of *p* < 0.05 was adopted; *p*-values between 0.05 and 0.1 were considered to indicate statistical trends.

## 3. Results

### 3.1. General Characteristics of the Handlers

The average age of the handlers was 44 years. The dogs lived in family environments consisting, on average, of three people, including one child, and multiple companion animals (typically 2 dogs and 2 cats). In most households, the dog had access to a garden and/or a terrace and was managed exclusively by the handler (87.5%; Table S3). For the majority of handlers, this was their first time training a dog for search work (n = 26, 83.5%), although they reported an average of four years of experience (range: 1–12 years; Table S3).

More than 80% of handlers reported that their motivation to become a SAR volunteer stemmed from a desire to help others, and over half of them also cited a passion for dog training (Table S3). All participants expressed moderate (score 3/5: n = 10, 31.3%) to high (score 4/5: n = 11, 34.4%; score 4/5: n = 11, 34.4%) level of satisfaction with their role.

### 3.2. Demographics, Behavioral History, Current Management, and Training of the SAR Dogs

Data related to the dogs’ characteristics and management are presented in Table S1. The sample comprised 14 females and 18 males, with the majority of dogs (n = 22) aged 1–4 years. Over 70% of the dogs were intact. The most represented breed was the Border Collie, followed by mixed-breed dogs, Belgian Malinois, and German or Working Line German Shepherd (German Shepherd Sable). Body weight ranged from 14 to 39 kg (mean = 24.9 kg). The relevant current or past medical conditions reported included: unilateral deafness, cauda equina syndrome, a previous episode of acute kidney failure, and one case of cruciate ligament surgery performed a few years earlier.

More than half of the dogs came from breeders. Notably, in one case, the dog had been adopted after being abandoned for unknown reasons and two handlers suspected that their dog had experienced mistreatment prior to adoption. Approximately 40% of the dogs had been initially acquired for companionship or for purposes unrelated to search work. On average, the dogs came from litters of 7 puppies (range: 4–11), and in nearly half of the cases, the puppy was selected based on the breeder’s recommendation, whereas only two handlers reported choosing their puppy based on observed temperament. The average age at adoption was 4 months (range: 60–1095 days).

More than 50% of handlers reported that their dogs’ daily routine was variable, and in four cases, the feeding schedule also varied. Only three dogs did not have access to the house. In most cases, when left alone, dogs were free to access either the garden or the house. More than 30% of dogs were allowed to sleep in the bedroom at night, and only two slept in a dog crate.

A total of 68.8% of handlers reported walking their dog for at least 30 min per day, most often off-leash. Seventeen percent engaged in additional physical activities with their dog, such as jogging or cycling, and 21% of dogs had the opportunity to play daily with other dogs. More than 80% of dogs showed clear signs of enthusiasm when anticipating time spent with their handler, such as appearing happy, wagging their tails, showing excitement, or displaying other joyful behaviors.

Details of the training-related responses are presented in Table S4. On average, dogs began training at 3 years of age (range: 1–7 years). Nineteen percent trained for less than 2 h per week, 32% trained between 2 and 5 h, and 48% trained for more than 5 h per week. According to 40% of respondents, training sessions occurred more than once per week, while in 50% of cases, training was held weekly. Many dogs had experience with different types of training (i.e., 16% with clicker training, 23% with obedience), and 25% had attended puppy classes. Handlers most commonly described their dogs’ behavior when anticipating a training session as excited (9 answers), happy (9 answers), focused (7 answers), curious (5 answers), attentive (4 answers), impatient (4 answers), motivated (4 answers), ready (4 answers), playful (2 answers), hyperactive (2 answers), and energetic (2 answers).

### 3.3. Dog Personality

#### 3.3.1. Adjectives Freely Provided by Handlers: Descriptive Statistics

The most frequently mentioned terms used by handlers to describe their dogs were: docile (n = 6), well-balanced (n = 5), intelligent (n = 5), beautiful (n = 4), curious (n = 4), sociable (n = 4), affectionate (n = 3), sweet (n = 3), clever (n = 3), playful (n = 3), motivated (n = 3), and high drive (n = 3). Figure S2 shows the resulting word cloud.

#### 3.3.2. Descriptor Scores Expressed on a Likert Scale: Multivariate Analyses and Associations Between Behavioral Traits and Dog–Handler Characteristics

**Figure 4:**
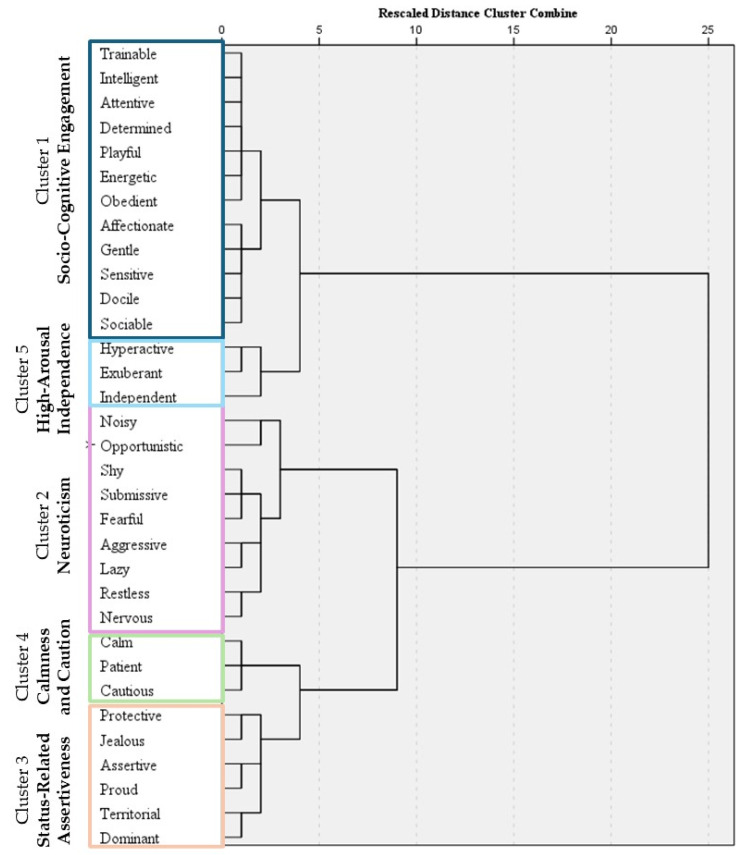
Dendrogram resulting from the hierarchical cluster analysis of the 33 behavioral descriptors rated on a Likert scale. The five clusters, identified based on the rescaled linkage distance, are highlighted with colored boxes (each in a different color), representing groups of variables with similar response patterns.

Table S5 shows the medians obtained for the 33 descriptors. The highest scores (i.e., 5) were recorded for *playful*, *intelligent*, *docile*, *sociable*, *affectionate*, and *energetic*, while the lowest scores (0) were for *aggressive* and *lazy*. All the descriptors expressed using a Likert scale were subsequently subjected to HCA. Based on the dendrogram (Figure 4), the following clusters of variables were identified:

- **Cluster 1 (Socio-Cognitive Engagement)** included the traits *trainable*, *intelligent*, *attentive*, *determined*, *playful*, *energetic*, *obedient*, *affectionate*, *gentle*, *sensitive*, *docile*, and *sociable*. This cluster reflects a combination of cognitive involvement, emotional responsiveness, and social affiliation, and was intended to capture a cooperative, adaptable, and affiliative behavioral profile. All items were coded such that higher scores indicated greater socio-cognitive engagement.
- **Cluster 2 (Neuroticism)** included the traits *noisy*, *opportunistic*, *shy*, *submissive*, *fearful*, *aggressive*, *lazy*, *restless*, and *nervous*. Neuroticism was conceptualized as a higher-order construct describing an individual’s tendency to experience negative emotional states and heightened reactivity to adverse or challenging situations. Consistent with canine personality research, these traits cluster around emotional insecurity, increased stress sensitivity, and impaired emotional regulation. These characteristics may manifest in various stress-related behaviors, ranging from fear and anxiety to aggression [28,37]. From an operational search and rescue perspective, this neurotic profile contrasts with resilience, which in dogs reflects emotional regulation, inhibitory control, and adaptability, key capacities for maintaining behavioral stability under demanding field conditions [2]. Within this framework, all items were considered complementary stress-related response patterns reflecting the same underlying disposition rather than opposite traits. Accordingly, all items were coded in the same direction [28], with higher scores indicating greater expression of the neurotic profile.
- **Cluster 3 (Status-Related Assertiveness)** comprised the traits *protective*, *jealous*, *assertive*, *proud*, *territorial*, and *dominant*. This cluster captured traits related to social control, resource guarding, and status-oriented interactions. All items were coded to reflect increasing expression of status-related assertiveness.
- **Cluster 4 (Calmness and Caution)** included *calm*, *patient*, and *cautious*. This cluster was intended to reflect low arousal and a deliberate, restrained response to environmental stimuli. In this context, caution was interpreted as thoughtful and controlled behavior rather than fearfulness, and all items were coded in the same direction, with higher scores indicating greater calmness and behavioral restraint.
- **Cluster 5 (High-Arousal Independence)** included the traits *hyperactive*, *exuberant*, and *independent*, representing high arousal, behavioral spontaneity, and a tendency toward independent or self-directed engagement with the environment. Higher scores reflected greater expression of this behavioral profile.

Cluster scores showed associations with specific demographic and management variables. In particular, Cluster 1 (Socio-Cognitive Engagement) was positively associated with the number of children in the household (ρ = 0.676, *p* < 0.05).

Cluster 2 (Neuroticism) was associated with the daily walk duration (F (2,29) = 6.15, *p* = 0.006), with dogs walking less than one hour per day exhibiting higher scores than those walking for more than one hour (*p* = 0.002), and was higher in dogs aged 2 to 6 years compared to younger dogs (F (2,29) = 2.25, *p* = 0.048).

Cluster 4 (Calmness and Caution) was negatively correlated with the number of years of experience as a search and rescue handler (ρ = −0.356, *p* < 0.05) and with the level of satisfaction of expectations in search and rescue activities (ρ = −0.406, *p* < 0.05).

### 3.4. Search Performance

In successful trials (78%), the mean time to locate the figurant was 5 min (median = 6; range: 2–12 min). Table 1 reports the descriptive statistics and inter-rater reliability for each behavioral parameter. Parameters with median scores below 4 included *Control*, *Distraction*, *Search Pattern*, *Confidence*, *Accuracy*, and *Independence*. No significant differences were found among the performance parameters assessed within individual dogs, suggesting consistent performance across the behavioral dimensions (Friedman χ^2^ (11)= 12.37, *p* = 0.337).

The analysis of inter-rater agreement showed that, for the parameters *Control*, *Distraction*, *Search Pattern*, and *Confidence*, agreement between instructor and handler ranged from none to fair (Kα ≤ 0.40). With the exception of *Distraction*, handlers consistently assigned higher scores than instructors for these parameters. For all remaining parameters, inter-rater agreement was moderate to substantial (Kα > 0.40).

Additional performance indicators were derived from GPS data (Table S6). The dogs’ average speed during searches was approximately 2 km/h, whereas the mean maximum speed was 23.2 km/h, with peak values of 41.1 km/h. Notably, 50% of the dogs reached speeds above 22.2 km/h, and 25% of the dogs exceeded 28.2 km/h. The altitude difference along the search paths averaged 37 m, ranging from 1 to 99 m. The average distance covered per session was 0.80 km, ranging from 0.13 to 2.51 km. The *Ground Exploration Index* had a mean value of 125 m/min, with a maximum of 460 m/min.

### 3.5. Associations Among Environmental Factors, Handler and Dog Characteristics, GPS Metrics, Behavioral Traits, and Performance During Search Tasks

**Figure 5:**
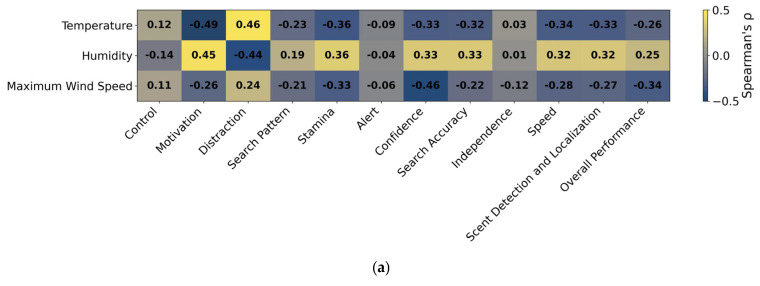

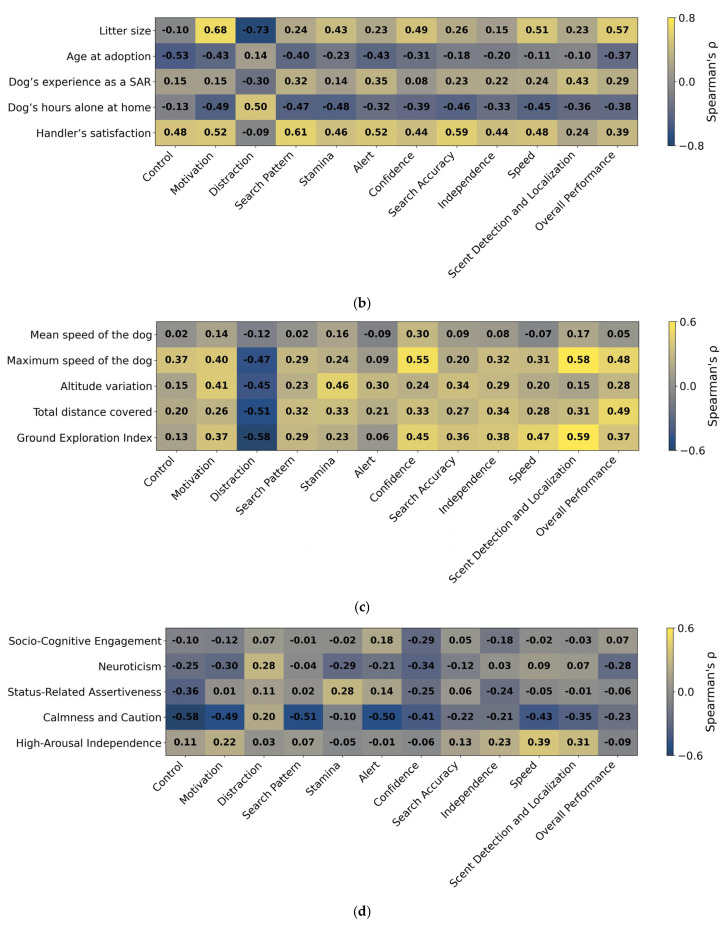
Heatmaps showing Spearman’s rank correlation coefficients (ρ) between performance scores and environmental parameters (**a**), dog and handler characteristics (**b**), parameters derived from GPS data (**c**), and behavioral traits derived through cluster analysis (**d**). Only parameters with │ρ│ ≥ 0.3 or relevant items are shown. Yellow hues indicate positive correlations, blue hues indicate negative correlations, while color intensity reflects the strength of the correlation. Heatmap visualizations were generated using AI tools (OpenAI’s ChatGPT, GPT-5). Individual *p*-values are reported in the Supplementary Material.

Correlations between environmental parameters, handler and dog characteristics, and performance scores are shown as heat maps (Figure 5), while conventional correlation matrices including individual *p*-values are reported in the Supplementary Material (Tables S7–S10).

Temperature (Figure 5a and Table S7) was significantly negatively associated with scores for *Motivation* and *Stamina*, while it was positively associated with the *Distraction* score. Conversely, humidity was positively associated with scores for *Motivation* and *Stamina* and negatively with the *Distraction* score. Maximum wind speed was negatively correlated with *Confidence* and *Overall performance* (for all: *p* < 0.05).

Performance scores were then analyzed to explore their potential associations with handler- and dog-related characteristics (Figure 5b and Table S8). Litter size was significantly positively associated with *Motivation*, *Stamina*, *Confidence*, *Speed*, and *Overall Performance*, and negatively associated with *Distraction* score. Age at adoption was significantly negatively associated with the *Control* score, *Motivation*, *Search Pattern*, *Alert*, and *Overall Performance*, while the dog’s experience as a SAR dog was positively associated with *Scent Detection* and *localization*. No other dog characteristics, including breed, were correlated with performance scores. Regarding dog management, the number of hours the dog was left alone at home was significantly positively correlated with several performance scores. Specifically, a positive association was observed with *Distraction*, whereas most of the other performance parameters showed strong or moderate negative associations with the duration of time the dog spent alone at home. The handler’s satisfaction score regarding their expectations in search and rescue work showed significant positive associations with *Control*, *Motivation*, *Search Pattern*, *Stamina*, *Alert*, *Confidence*, *Search Accuracy*, *Independence*, *Speed*, and *Overall Performance* (for all: *p* < 0.05). All remaining parameters showed poor or not significant associations with performance.

Regarding the parameters recorded via GPS (Figure 5c and Table S9), maximum speed was significantly positively associated with *Control*, *Motivation*, *Confidence*, *Scent detection* and *localization*, and *Overall performance*, while it was negatively correlated with *Distraction*. The altitude difference covered during the trial was significantly positively associated with *Motivation* and *Stamina*, while it was negatively associated with *Distraction*. The distance covered by the dog was positively associated with *Overall performance*, while it was negatively correlated with *Distraction*. Finally, the Ground Exploration Index was significantly positively correlated with *Confidence*, *Independence*, *Speed*, *Scent Detection* and *Localization*, and *Overall Performance*, while it was negatively correlated with *Distraction* (for all: *p* < 0.05).

Analyses also revealed some associations involving behavioral trait clusters (Figure 5d and Table S10). Cluster 3 (Status-Related Assertiveness) showed significant negative associations with *Control*. Cluster 4 (Calmness and Caution) was negatively correlated with *Control*, *Motivation*, *Search Pattern*, *Alert*, *Confidence*, *Speed*, and *Scent Detection* and *localization*, while Cluster 5 (High-Arousal Independence) was positively correlated with *Speed*.

It should be noted that, after FDR correction, none of the associations remained statistically significant. This conservative outcome was expected, given the large number of variables tested and the resulting substantial lowering of the significance threshold imposed by the FDR correction. However, given the associated risk of Type I error, these correlation analyses are best regarded as exploratory and indicative of potential trends rather than confirmatory relationships.

## 4. Discussion

This study represents the first attempt to simultaneously examine how environmental conditions, behavioral history, management practices, and personality traits may influence search dog performance in a simulated operational context. It also introduces an innovative methodology that integrates subjective behavioral ratings with objective GPS-derived performance metrics, enabling a comprehensive assessment of key factors relevant to SAR dog operations.

Another distinctive feature of this study is the involvement of volunteer handlers rather than military professionals, which likely explains why some management- and dog-related aspects differ from those reported in the literature [6,38,39]. Many dogs were originally adopted as companions, resulting in greater variability in breed, background, and life history, including abandonment or suspected mistreatment. Unlike military working dogs, generally housed individually and managed under highly standardized routines [20,38,39], the dogs in this study were managed in ways more closely resembling companion animal care. Most had access to a garden, were allowed to move freely within the house, slept in the bedroom, and lived as family members with children and other pets. However, behavioral profiles extracted from the multivariate analysis reflected traits typical of working dogs: the first cluster (Socio-Cognitive Engagement) primarily emphasized trainability, cognitive engagement, and cooperativeness, whereas other clusters were characterized by assertiveness and arousal. Thus, despite pet-like management practices, volunteer handlers valued cognitive aspects of sociability that are more directly functional for operational performance, rather than primarily emotional or affiliative traits emphasized in companion animal management [40,41].

Svartberg [8] identified similar dimensions in working dogs and grouped them into a higher-order factor, interpreted as a shyness–boldness continuum. The present study retained all extracted dimensions to preserve a more detailed characterization of behavioral traits. This approach allowed us to highlight, for example, that living in a family environment with children was associated with higher scores in the Socio-Cognitive Engagement trait, suggesting a link between socially stimulating environments and dogs’ cognitive and social characteristics [28,42,43]. Unexpectedly, greater handler experience and higher satisfaction with rescue work were associated with dogs showing lower levels of calmness and caution. This pattern may reflect a preference for more energetic and proactive coping styles, traits that, as explored below, are indeed associated with stronger operational performance.

All aspects related to the characteristics of both dogs and handlers were then associated with the dogs’ actual search performance. Performance was evaluated using the behavioral scoring system proposed by Clark, Rooney, and colleagues [24,25], which appeared to be intuitive, quick to complete, and practical for field use. The most challenging parameter was *Distraction*, likely because, unlike the others, higher scores on the evaluation form corresponded to poorer performance. This inconsistency likely confused instructors and, in particular, handlers, thereby contributing to low inter-rater agreement. Reframing this parameter as “*Absence of Distraction*” may improve clarity and scoring consistency. Low agreement between instructors and handlers was also observed for *Control*, *Search Pattern*, and *Confidence*, with handlers generally overestimating their dogs’ abilities. This tendency aligns with Clark et al. [24], who found that handlers tended to rate their own dogs more favorably than impartial raters. This self-report bias was expected and may have several explanations. Handlers may be reluctant to assign low scores to avoid reflecting poorly on their own performance, particularly for the parameter *Control*. This could stem from a sense of ‘loyalty,’ comparable to friendship bias, as may be the case for the parameter *Confidence*. Handlers may also be influenced by prior expectations of their dogs’ abilities [24,44]. Finally, given the variability in handler experience, some handlers may have lacked the competence or knowledge required to accurately self-assess certain parameters, such as *Search Pattern*. Although future studies could use more homogeneous samples, these findings highlight the importance of comprehensive handler training to improve understanding of the behavioral components underlying effective SAR performance.

Conversely, the instructors’ extensive experience ensured robust evaluations, as evidenced by substantial correlations between their scores and objective GPS data. Dogs showing greater speed and area coverage received higher scores for efficiency and motivation. Conversely, they scored lower on *Distraction*, indicating superior attentional control. This combination of endurance and cognitive focus aligns with previous studies [6,11,45]. These findings confirm that performance is determined by more than just olfactory ability; it also depends on proactive engagement and regulation. Specifically, dogs that exhibited greater speed, wider exploratory behavior, and a proactive search strategy performed better in the field.

The impact of environmental parameters on performance scores was also assessed. Increasing temperature was negatively correlated with *Motivation* and *Stamina*, whereas it was positively correlated with *Distraction*. This decline in performance likely stems from physiological and chemical mechanisms. Higher temperatures trigger metabolic and thermoregulatory responses that reduce stamina [46] and increase panting, which diverts airflow from the olfactory recess and decreases sniffing rate and olfactory efficiency [7,41,47]. Temperature also alters the volatility and chemical structure of VOCs, as well as bacterial activity and skin secretions, modifying scent rafts and odor signatures [2]. Notably, these associations emerged even within a moderate temperature range (13–24 °C). Our findings align with previous studies [48,49], while others reported no effect of temperature on localization performance [50,51]. Variations in methodology and search environments, such as the smaller testing area used by other authors [50], may account for these differences. In any case, given the risk of Type I error from the large number of correlation analyses conducted in this study, our findings should be interpreted as indicating potential patterns rather than definitive effects. Nevertheless, extreme temperatures are also associated with an increased risk of heat-related illnesses [52]. Accordingly, SAR dog operations should always be organized with careful consideration of the multiple adverse effects of temperature on both performance and canine welfare.

In contrast, humidity tended to show a positive association with performance, likely by preventing nasal dryness and preserving scent plumes [53]. It also affects human odor sources, for instance, by increasing aerosol particle growth and accelerating ammonia formation [2]. Previous studies on humidity have reported mixed effects [4,11]. However, because excessive moisture can elevate heat stress risks [54], moderate humidity likely provides the most favorable conditions for SAR dogs.

Finally, a higher maximum wind speed was associated with reduced *Confidence* and resulted in lower overall performance. Wind influences odor transport through advection, dispersion, turbulence, and gravitational forces acting on VOCs, skin rafts, and aerosols [2]. Strong winds may amplify turbulence and dispersion, diluting odor concentration and making trails difficult to interpret. Such conditions could confuse the dog and induce frustration, explaining the reduced scores for *Confidence* and *Overall performance* indicators. Our findings are consistent with those of Jinn et al. [5], whereas Reed et al. [55] found no wind effect, possibly due to confounding rainfall effects. Although dogs can partially exploit wind direction through adaptive movement [21] and wind is essential for odor plume formation and transport [56], very high wind speeds can excessively mix and disperse odor cues, thereby impairing reliable odor tracking.

Performance was also examined in relation to dog characteristics. Consistent with previous research [6,8,57], no association was found between breed and search performance. Overall, our results suggest that the influence of personality, social, and contextual factors on search performance may outweigh breed-specific genetic differences. Specifically, litter size emerged as a potential predictor. In the present sample, dogs from larger litters displayed higher motivation, focus, resilience, and overall performance. This finding is consistent with the classic work of Scott and Fuller [58], which demonstrated the long-term effects of early developmental experiences on adult canine behavior. More recent evidence further indicates that litter-related factors explain a greater proportion of behavioral variability than breed (~23% vs. 10%) [59]. Puppies raised in larger litters tend to display greater activity and exploratory behavior, possibly as an adaptation to early-life competition for maternal resources [60]. Such early social challenges may foster persistence and stress-coping abilities, traits that later enhance performance in tasks requiring sustained motivation, focus, and adaptability. From an applied perspective, litter size and early social environment may therefore represent useful indicators of working potential.

Better performance, both in terms of control and motivation/strategy scores, was also associated with an earlier age at adoption. Early adoption may allow handlers to shape the dog’s development from the outset, facilitating exposure to relevant environments and stimuli encountered during work, while strengthening the dog–handler bond. Although separation from the mother and littermates should not occur before 7–9 weeks of age [21], timely adoption can enrich the socialization period in working dogs through gradual exposure to equipment (e.g., harness handles), sounds, and environmental challenges [21,61]. Early adoption may also be important to foster the proper rebuilding of attachment from the mother to the handler, thereby promoting the early development of a constructive human–dog relationship. It is not surprising, therefore, that our results also confirm the effectiveness of training and highlight the importance of experience as a SAR dog, which primarily influenced the performance scores related to search methodology and accuracy.

As mentioned above, a high level of handler satisfaction was also associated with better performance. It is intuitive that the most satisfied handlers are those who know their dogs work well. However, the reverse has also been demonstrated [62]. It can be hypothesized that a satisfied and confident handler may positively influence the dog’s performance, potentially through a classic *Clever Hans* effect. Interestingly, the negative association between time spent alone at home and search performance further highlights the central role of the dog–handler relationship. Limited interaction with the handler outside training may reduce opportunities to strengthen mutual understanding, trust, and communication, key elements for successful cooperation in SAR operations [6,20,63]. Thus, the frequency and quality of daily dog–handler interactions appear to influence not only dogs’ welfare but also operational efficiency. Viewed collectively, our results emphasize the importance of both the early socialization phase within the litter and the secondary socialization phase, which encompasses bonding and social conditioning within the new environment, the future working context, and, last but not least, with the handler.

The final step of the analysis examined the relationship between search performance and behavioral traits. Dogs scoring highly on behavioral traits indicative of calmness, caution, and restraint tended to show lower *Motivation* and *Confidence*. Crucially, they also displayed poorer *Control* and *Search accuracy*. In contrast, dogs characterized by high arousal, energy, and autonomy achieved the best performance. This aligns with previous research [6,8] and perspective studies [10,11,12] identifying high energy levels as advantageous for search work. Troisi et al. [41] described the relationship between arousal and performance as curvilinear, with excessive arousal potentially leading to anxiety and distress in scent detection dogs. The present study did not capture the descending phase of this curve (i.e., the decline in performance at very high levels of arousal) and lacked tools to assess distress; thus, the relationship appears linear. Similarly, Diverio et al. [6] described these traits in avalanche search dogs in terms of proactive *versus* passive coping styles, while Svartberg [8] conceptualized them along the shyness–boldness axis. In the present study, the traits most strongly associated with performance appear to align along a high–low reactivity dimension, extending from arousal, general activity, exuberance, and independence to calmness, caution, and self-restraint.

As with all empirical research, this study has several limitations. Surface search conditions precluded video recording, limiting detailed behavioral analyses; however, this constraint reflects the realities of large and complex outdoor field simulations. Moreover, the use of multiple search areas and hiding locations, while necessary to reduce handler familiarization with the search environment, may have introduced additional environmental variability. Findings on breed effects remain inconclusive, likely due to self-report bias in handler questionnaires and the high breed variability, which reduced the effective sample size for each group. In addition, although several associations were observed at the level of individual analyses, none remained statistically significant after correction for multiple testing. This loss of significance is likely attributable, at least in part, to the limited sample size, which constrained statistical power and the ability to detect small-to-moderate effects in a field-based context. Future studies should optimize these aspects and incorporate physiological indicators of distress. Additionally, including measures of the handler–dog relationship could help clarify its influence on handler perception and dog performance.

## 5. Conclusions

Search performance appears to be a multifactorial construct shaped by the interaction of extrinsic factors, such as environmental conditions, and intrinsic factors, including personality, early life experiences, as well as social and contextual influences. Systematic monitoring of environmental conditions should therefore be incorporated into the organization of dogs’ work schedules to optimize individual performance while safeguarding welfare. In addition, careful management of puppies’ sensitive developmental phases, together with motivation-oriented training pathways for both handlers and dogs, may help maintain high performance levels.

## Figures and Tables

**Table 1:** Descriptive statistics and inter-rater reliability indices for behavioral parameters (mean difference, Kα = Krippendorff’s α with 95% CI from 1000 bootstrap samples) between instructor and handler. Coefficients indicating none-to-fair agreement (α ≤ 0.40) are in bold.

Behavioral Parameters	Median and Range *	Mean Score		Mean Difference	Kα	95% CI
		Instructor	Handler			
Control	3 (1–5)	3.4	4.0	−0.674	**0.139**	−0.311, 0.471
Motivation	4 (1–5)	3.7	4.0	−0.348	0.437	−0.009, 0.750
Distraction	3 (1–5)	2.7	2.0	0.727	**0.143**	−0.287, 0.491
Search Pattern	3 (1–5)	3.2	3.8	−0.546	**0.275**	−0.195, 0.594
Stamina	4 (1–5)	3.7	3.5	0.091	0.462	0.080, 0.721
Alert	4 (1–5)	3.5	3.8	−0.273	0.610	0.273, 0.797
Confidence	3 (1–5)	3.3	4.3	−0.857	**0.287**	−0.163, 0.600
Search Accuracy	3 (1–5)	3.4	4.0	−0.546	0.505	0.088, 0.785
Independence	4 (1–5)	3.5	3.8	−0.341	0.553	0.183, 0.788
Speed	4 (1–5)	3.5	3.8	−0.326	0.652	0.318, 0.819
Scent Detection and Localization	4 (1–5)	3.7	4.0	−0.227	0.571	0.173, 0.760
Overall Performance	4 (1–5)	3.4	3.8	−0.409	0.527	0.039, 0.807

* assigned by the instructor.

## Data Availability

The original contributions presented in this study are included in the article/Supplementary Material. Further inquiries can be directed to the corresponding authors.

## Supplementary Materials

The following supporting information can be downloaded at: https://www.mdpi.com/article/10.3390/ani16040664/s1, Table S1. Demographic, origin, socialization, and management variables of dogs. Table S2. Environmental variables recorded by the weather station during the trials. Table S3. General characteristics of handlers including their prior experience and motivations for engaging in search and rescue work. Table S4. Descriptive summary of the types of training received by the dogs. Table S5. Descriptors sorted by median score (from highest to lowest), along with the first quartile (Q1) and third quartile (Q3) for each descriptor. Table S6. Descriptive statistics of variables recorded by the GPS device during the trials. Table S7. Spearman’s rank correlation coefficients between performance evaluation scores and environmental parameters collected via weather station. Table S8. Spearman’s rank correlation coefficients between performance evaluation scores and dog and handler characteristics. Table S9. Spearman’s rank correlation coefficients between performance evaluation scores and GPS-derived parameters. Table S10. Spearman’s rank correlation coefficients between performance evaluation scores and behavioral traits derived through cluster analysis. Figure S1. Questionnaire used to collect information on handler and dog demographics, management practices, and personality traits. Figure S2. Word cloud displaying the most frequently mentioned descriptors.
