# Supplementary material for: What Makes Canine Search and Rescue Successful? Insights into Environmental, Management, and Personality Factors
Source: Animals (Basel). 2026 Feb 19;16(4):664. doi: 10.3390/ani16040664 (PMC12937267; doi:10.3390/ani16040664)

Supplementary materials

Table S1. Demographic, origin, socialization, and management variables of dogs. Results are presented as absolute (N°) and relative (N %) frequencies.

Variable	Category	N°	N %
Sex	Female	14	43.8%
	Male	18	56.3%
Age (categorized)	< 1 year	1	3.1%
	1–<2 years	6	18.8%
	2–<3 years	6	18.8%
	3–<4 years	10	31.3%
	4–<5 years	3	9.4%
	5–<6 years	4	12.5%
	≥ 6 years	2	6.3%
Gonadectomy	No	20	71.4%
	Yes	8	28.6%
Age at gonadectomy	>1 year	2	25.0%
	≤ 1 year	6	75.0%
Breed	Australian Kelpie	1	3.1%
	Border Collie	7	21.9%
	Golden Retriever	1	3.1%
	Labrador Retriever	3	9.4%
	Belgian Malinois	5	15.6%
	Mixed-breed	6	18.8%
	Mudi	1	3.1%
	Nova Scotia Duck Tolling Retriever	1	3.1%
	Australian Shepherd	2	6.3%
	German Shepherd Dog	2	6.3%
	Working Line German Shepherd	2	6.3%
	Weimaraner	1	3.1%
Daily routine	Fixed	14	43.8%
	Variable	18	56.3%
	Yes	20	64.5%
Dog's living environment	Indoors (house)	15	46.9%
	Both indoors and outdoors	14	43.8%
	Outdoors (garden)	3	9.4%
Where the dog is kept when left alone	Outdoor kennel	2	8.0%
	Free in the yard	6	24.0%
	Free in the house (daytime)	12	48.0%
	Confined to a room	2	8.0%
	Transport crate	3	12.0%
Where the dog stays at night	In the bedroom	8	34.8%
	Outdoors	7	30.4%
	Free in the living area	6	26.1%
	In a transport crate	2	8.7%

Source of acquisition	Breeder	17	56.7%
	Private individuals	11	36.7%
	Associations	1	3.3%
	From owner's own dogs	1	3.3%
Main reason for acquiring the dog	Companionship for myself	1	3.2%
	Companionship for the family	10	32.3%
	Search work	19	61.3%
	To rescue/save the dog	1	3.2%
Criteria for selecting the puppy from the litter	Based on appearance	7	31.8%
	Chosen by the breeder	10	45.5%
	Chosen by the breeder and for being the most outgoing	1	4.5%
	For being the most outgoing	1	4.5%
	No specific selection criteria	3	13.6%
Feeding frequency	More than twice per day	2	6.3%
	Once per day	5	15.6%
	Twice per day	25	78.1%
Feeding schedule	Regular	28	87.5%
	Variable	4	12.5%
Daily walk duration	Does not go for walks	4	12.5%
	Less than 15 minutes	1	3.1%
	15–30 minutes	5	15.6%
	30–60 minutes	9	28.1%
	1–2 hours	6	18.8%
	3–4 hours	7	21.9%
How the dog is walked	Choke collar	2	6.3%
	Leash	7	21.9%
	Off-leash	21	65.6%
	Harness	2	6.3%
During walks, how much of the time does the dog spend off-leash (proportionally)?	Never	4	12.5%
	Less than 25%	4	12.5%
	25-50%	8	25.0%
	50-75%	7	21.9%
	75-100%	9	28.1%
Do you engage in physical activity with your dog?	No	23	82.1%
	Yes	5	17.9%
Frequency of play sessions with other dogs	Never	4	13.8%
	Monthly- Every two weeks	6	20.6%
	Weekly	11	37.9%
	Occasionally	2	6.9%
	Daily	6	20.7%

Table S2. Environmental variables recorded by the weather station during the trials. Parameters include mean temperature, humidity, wind speed, and atmospheric pressure.

Parameter	Mean	Standard Deviation	Median	Minimum	Maximum	Percentile 25	Percentile 75
Temperature (mean, °C)	19.53	3.12	20.40	13.20	24.10	17.30	21.85
Humidity (mean, %)	63.85	12.61	60.00	45.50	86.00	56.50	74.00
Maximum Wind Speed (Km/h)	6	5	3	0	15	2	12
Atmospheric pressure (hPa)	868.79	.84	869.00	867.20	870.20	868.34	869.30

Table S3. General characteristics of handlers including their prior experience and motivations for engaging in search and rescue work. Continuous variables are expressed as mean and range, while categorical variables are presented as absolute (N°) and relative (N %) frequencies.

Variable		N°	N %	Mean	Minimum	Maximum
Handler's age (years)		-	-	44	19	65
Family members (n°)		-	-	3	1	8
Number of adults in the household		-	-	2	1	7
Number of children in the household		-	-	1	1	3
Number of dogs in the household		-	-	2	1	5
Number of cats in the household		-	-	2	1	5
The family lives in a home with:	Apartment only	4	12.5%			
	Garden	15	46.9%			
	Garden and terrace	3	9.4%			
	Terrace	10	31.3%			
Who takes care of the dog?	Handler only	28	87.5%			
	Handler and a family member	3	9.4%			
	All family members	1	3.1%			
First search dog (yes/no)	No	5	16.1%			
	Yes	26	83.9%			
Years of experience as a rescuer				4	1	12
Motivation to become a SAR handler	Desire to help	26	81.3%			
	Passion for dogs/dog training	18	56.3%			
	Interest in canine behavior	12	37.5%			
	Emergency response interest	11	34.4%			
	Personal reasons	6	18.8%			
	Social engagement	5	15.6%			
	Skill development	9	28.1%			

Table S4. Descriptive summary of the types of training received by the dogs. Results are presented as absolute (N°) and relative (N %) frequencies.

Variable	Category	N°	N %
Weekly training duration (h)	0-2 h	6	19.4%
	2-5 h	10	32.3%
	> 5 h	15	48.4%
Frequency of search training	Daily	1	3.1%
	Several times per week	13	40.6%
	Weekly	16	50.0%
	Monthly	1	3.1%
	Every two weeks	1	3.1%
Primary search environment used for training	Woodland	15	46.9%
	Woodland and open field	13	40.6%
	Woodland, open field, and rubble field	2	6.3%
	Open field	1	3.1%
	Mixed environments	1	3.1%
Other training in addition to search training	Crate habituation	23	71.9%
	Puppy class	8	25.0%
	Obedience	7	22.6%
	Agility	3	9.4%
	Clicker training	5	15.6%
	Dog shows	1	3.1%
	Pet therapy	1	3.1%

Table S5. Descriptors sorted by median score (from highest to lowest), along with the first quartile (Q1) and third quartile (Q3) for each descriptor.

Descriptor	Median	Percentile 25	Percentile 75
Playful	5	4	5
Intelligent	5	4	5
Docile	5	4	5
Sociable	5	3	5
Affectionate	5	4	5
Energetic	5	4	5
Sensitive	4	3	5
Exuberant	4	3	5
Obedient	4	3	5
Trainable	4	4	5
Attentive	4	4	5
Determined	4	3	5
Protective	3	1	5
Jealous	3	2	4
Calm	3	2	4
Hyperactive	3	1	4
Independent	3	2	4
Dominant	3	1	4
Proud	3	2	3
Gentle	3	3	5
Fearful	2	1	3
Territorial	2	1	3
Patient	2	1	4
Assertive	2	1	3
Cautious	2	1	4
Opportunistic	2	1	3
Noisy	1	0	3
Restless	1	1	2
Nervous	1	0	2
Submissive	1	0	2
Shy	1	0	1
Aggressive	0	0	1
Lazy	0	0	1

Table S6. Descriptive statistics of variables recorded by the GPS device during the trials.

Parameter	Mean	Standard Deviation	Median	Minimum	Maximum	Percentile 25	Percentile 75
Average speed of the dog (km/h)	1.93	1.06	1.60	0.70	4.30	1.15	2.75
Maximum speed of the dog (km/h)	23.21	8.32	22.20	11.10	41.10	16.55	28.15
Difference in altitude (m)	37	27	34	1	99	18	44
Distance covered by the dog (km)	0.80	0.63	0.56	.13	2.51	0.40	0.90
Time in movement (min)	19	14	15	8	68	11	19
Ground Exploration Index (m/min)	125	109	106	7	460	52	152

Table S7. Spearman's rank correlation coefficients between performance evaluation scores and environmental parameters collected via weather station. Medium and large significant ($p \leq 0.05$) correlations are indicated in bold. **$p \leq 0.01$, *$p \leq 0.05$, $^{\#}p < 0.1$.

	Control	Motivation	Distraction	Search Pattern	Stamina	Alert	Confidence	Search Accuracy	Independence	Speed	Scent Detection and Localization	Overall Performance
Temperature	0.124	**-0.493***	**0.463***	-0.234	**-0.365***	-0.089	-0.327#	-0.324#	0.032	-0.337#	-0.333#	-0.258
Humidity	-0.139	**0.452***	**-0.443***	0.189	**0.363***	-0.036	0.333#	0.329#	0.007	0.316#	0.324#	0.249
Maximum Wind Speed	0.109	-0.262	0.242	-0.215	-0.325#	-0.060	**-0.456***	-0.219	-0.121	-0.279	-0.272	**-0.336***

Table S8. Spearman’s rank correlation coefficients between performance evaluation scores and dog and handler characteristics. Medium and large significant ($p \le 0.05$) correlations are indicated in bold. $^{**}p \le 0.01$, $^{*}p \le 0.05$, $^{\#}p<0.1$.

	Control	Motivation	Distraction	Search Pattern	Stamina	Alert	Confidence	Search Accuracy	Independence	Speed	Scent Detection and Localization	Overall Performance
Litter size	-.095	**.684****	**-.727****	.244	**.429***	.232	**.487***	.263	.147	**.507***	.234	**.567***
Age at adoption	**-.529****	**-.427***	.145	**-.400***	-.234	**-.426***	-.312$^{\#}$	-.176	-.200	-.113	-.102	**-.369***
Dog’s experience as a SAR	.153	.149	-.298	.324$^{\#}$	.139	.346$^{\#}$	.076	.229	.224	.244	**.426***	.293
Dog’s hours alone at home	-.130	**-.493***	**.501***	**-.469***	**-.484***	-.322$^{\#}$	**-.388***	**-.459***	-.330$^{\#}$	**-.453***	**-.361***	**-.380***
Handler’s satisfaction	**.483****	**.517****	-.092	**.612****	**.457***	**.523****	**.442***	**.590****	**.439***	**.480***	.236	**.387***

Table S9. Spearman's rank correlation coefficients between performance evaluation scores and GPS-derived parameters. Medium and large significant ($p \leq 0.05$) correlations are indicated in bold. **$p \leq 0.01$, *$p \leq 0.05$, #$p < 0.1$.

	Control	Motivation	Distraction	Search Pattern	Stamina	Alert	Confidence	Search Accuracy	Independence	Speed	Scent Detection and Localization	Overall Performance
Mean speed of the dog	0.017	0.138	-0.119	0.021	0.157	-0.091	0.296	0.094	0.080	-0.069	0.169	0.050
Maximum speed of the dog	**0.368***	**0.402***	**-0.475***	0.289	0.243	0.089	**0.546****	0.203	0.318#	0.308#	**0.584****	**0.478***
Altitude variation	0.152	**0.414***	**-0.446***	0.227	**0.458***	0.296	0.240	0.336#	0.290	0.198	0.149	0.281
Total distance covered	0.195	0.264	**-0.508***	0.323	0.326#	0.212	0.334#	0.274	0.344#	0.280	0.310#	**0.488***
Ground Exploration Index	0.132	0.368#	**-0.576****	0.291	0.233	0.057	**0.453***	0.363#	**0.384***	**0.467***	**0.591****	**0.374***

Table S10. Spearman's rank correlation coefficients between performance evaluation scores and behavioral traits derived through cluster analysis. Medium and large significant (p≤0.05) correlations are indicated in bold. **p≤0.01, *p≤0.05, #p<0.1.

	Control	Motivation	Distraction	Search Pattern	Stamina	Alert	Confidence	Search Accuracy	Independence	Speed	Scent Detection and Localization	Overall Performance
Socio-Cognitive Engagement	-.099	-.118	.066	-.012	-.019	.181	-.287	.048	-.181	-.023	-.032	.074
Neuroticism	-.253	-.299	.277	-.037	-.290	-.207	-.336#	-.124	.027	.087	.066	-.278
Status-Related Assertiveness	**-.359***	.008	.107	.018	.285	.144	-.249	.055	-.243	-.047	-.005	-.055
Calmness and Caution	**-.584****	**-.490***	.196	**-.511***	-.104	**-.503***	**-.411***	-.217	-.212	**-.434***	**-.351***	-.225
High-Arousal Independence	.111	.222	.025	.070	-.053	-.015	-.055	.127	.226	**.385***	.314#	-.093

Figure S1. Questionnaire Used to Collect Demographic Information, Behavioral History, Management, and Personality Data of Dogs and Handlers

SAR MANAGEMENT AND BEHAVIOR QUESTIONNAIRE

Place and Date______________________

OWNER INFORMATION

Name__________________________

Address__________________________ City_________________ Zip Code_________

e-mail____________________________________

Age: ________

Occupation: _________

Household members: _____________________

Number of adults in the household (>18 years)____

Number of children in the household ____ Children's ages________________

Total number of dogs_____

**Name**	**Breed**	**Sex**	**Age (months)**
Dog 1			
Dog 2			
Dog 3			

Number of cats _____

Number of other animals (specify species) ____________________________

Type of home:
- ○ with garden
- ○ with terrace
- ○ apartment without garden or terrace

Who primarily cares for the dog?
- ○ The Handler
- ○ A family member
- ○ Other ___________________________

Is this your first dog used for search activity?
- ○ No
- ○ Yes→ How many? Operational?______

1

How many years have you worked or aspired to work as a rescuer?_____________

What motivated you to start this activity? (answer with one sentence or a few keywords)

- ☐ Desire to help others
- ☐ Passion for working dogs
- ☐ Interest in dog behavior and training
- ☐ Interest in emergency services
- ☐ Personal experience
- ☐ Social connections / friendship
- ☐ Skill development
- ☐ Other (specify)__________

Have your expectations regarding this activity been met? (score from 1 = not at all satisfied to 5 = extremely satisfied)

1	2	3	4	5

In what ways and how would you like to improve your activity as a rescuer?
____
____
____
____

2

DOG – DEMOGRAPHIC INFORMATION AND EARLY-LIFE EXPERIENCES

Name: ____________________

Sex: ___________

Age: __________

Breed: ________________________

Body weight (kg): ________

BCS[1]: ____

Gonadectomy:
- ○ No
- ○ Yes → Age: years_____ months_____
- ○ I don't know

Current clinical conditions: ____
____

Previous medical conditions: ____
____

Current treatments: __________________________ dosage (mg/Kg) _______
__________________________ dosage (mg/Kg) _______

Origin:
- ○ Owned
- ○ Breeder
- ○ Pet shop
- ○ Associations/Rescue
- ○ Private individuals
- ○ Stray
- ○ I don't know
- ○ Other: _____________

Reason why the dog was obtained:
- ○ Search and rescue
- ○ Companionship for an adult
- ○ Family companionship
- ○ Children's companionship
- ○ Rescue from a difficult situation
- ○ Guard dog
- ○ Herding dog
- ○ Obedience
- ○ Hunting dog
- ○ Show dog
- ○ For breeding purposes
- ○ Assistance dog
- ○ Other: _______

Age at separation from the litter: ________________ ○ I don't know

Age at adoption: ______________________ ○ I don't know

Litter size[2]: _________ ○ I don't know

---

[1] Based on the handler's assessment, using a 5-point scale (1 = underweight, 5 = obese)
[2] Including the dog under evaluation

3

Besides the mother and littermates, were other dogs present?
- ○ Yes_______
- ○ I don't know

If obtained as a puppy:

a. Where was the dog raised before adoption?
- ○ In a house
- ○ In a garage
- ○ Free outdoors
- ○ Puppy mill/Intensive breeding
- ○ Other: _______
- ○ I don't know

b. How was the puppy chosen within the litter?
- ○ Selected by the breeder
- ○ No choice
- ○ The most outgoing
- ○ The shyest
- ○ The largest
- ○ The smallest
- ○ Based on appearance
- ○ Other:_____________

If previously owned by others:

a. Why was the dog originally acquired by the previous owners?
- ○ Companionship for an adult
- ○ Family companionship
- ○ Children's companionship
- ○ Show dog
- ○ For breeding purposes
- ○ Guard dog
- ○ Herding dog
- ○ Obedience
- ○ Assistance dog
- ○ Hunting dog
- ○ Search/training
- ○ Other: ________
- ○ I don't know

b. **What is the reason the dog was given up?**
- ○ Family/personal problems of the previous owner
- ○ Dog management problems
- ○ Dog aggression
- ○ Other behavioral problems
- ○ Other:___________
  ____
  ____

Do you suspect your dog suffered mistreatment before living with you? ○ Yes ○ No

4

DOG - MANAGEMENT

Diet	% Composition		Brand
○ Dry<br>○ Wet<br>○ Dry and wet<br>○ Home-cooked<br>○ Other ______________			____________<br>____________

Meal frequency:
○ 1/day ○ 2/day ○ >2/day ○ Ad libitum

Meal administration: ○ Regular ○ Variable

Food rewards: ○ Yes ○ No Type:____________
Contingent on behavior: ○ Yes ○ No

Average number of hours the dog is left alone during weekdays:__________

Daily routine: ○ Fixed ○ Variable

The dog lives:
○ Only indoors ○ Only in the garden ○ Both indoors and outdoors

Where is the dog kept when alone?

- ○ Crate/cage
- ○ In a room
- ○ Free in the living area
- ○ Garage
- ○ Basement
- ○ Outdoor kennel
- ○ Tied outside
- ○ Free in the yard
- ○ Other: ________

Where does the dog stay during the night?

- ○ Crate/cage
- ○ In a room
- ○ Free in the living area
- ○ Garage
- ○ Basement
- ○ Bedroom
- ○ On someone's bed
- ○ Outdoors
- ○ Other: ________

Weekly activities: ○ Are fixed ○ Vary week to week

5

DOG - PHYSICAL ACTIVITY

Duration of daily walks:

- ○ Lives in the garden, no walks
- ○ Less than 15 min
- ○ 15-30 min
- ○ 30-60 min
- ○ 1-2 hours
- ○ 3-4 hours
- ○ More than 5 hours

Do you do physical activity together like jogging or cycling? ○ Yes ○ No

During walks, the dog is:

- ○ Free (off-leash)
- ○ On leash (regular collar)
- ○ With choke chain
- ○ With harness
- ○ With head collar (halter)
- ○ With prong collar

Reason for choosing this equipment: ____

During walks, what proportion of time is spent without a leash:

○ Never ○ Less than 25% ○ 25-50% ○ 50-75% ○ 75-100%

Frequency of play sessions with other dogs:

○ Never ○ Monthly ○ Once every two weeks ○ Weekly ○ Daily

When the dog perceives that a walk is about to start, it appears: (describe with 3 terms)

1. ____
2. ____
3. ____

6

DOG - SEARCH TRAINING

The dog trains for search activities:

- ○ In rubble
- ○ In water
- ○ Mantrailing (scent dogs)
- ○ On surface
- ○ In avalanche
- ○ Other: ______________________

At what age did the dog start search training? Years:________Months:_____ ○ I don't know

How long has the dog been training for search? ________________________

The dog is operational for search:

- ○ In rubble
- ○ Mantrailing (scent dogs)
- ○ In water
- ○ Not operational
- ○ On surface
- ○ In avalanche
- ○ Other:_____________________

Training level:

- ○ Basic
- ○ Intermediate
- ○ Advanced

Weekly training duration:

- ○ 0-1 h
- ○ 1-2h
- ○ 2-3 h
- ○ 3-5 h
- ○ 5-7 h
- ○ 7-10 h
- ○ More than 10 h

Overall frequency of search training:

- ○ Never
- ○ Monthly
- ○ Every two weeks
- ○ Several times per day
- ○ Weekly
- ○ Daily
- ○ Several times per week

Main search environment used for training:

- ○ Woodland
- ○ Open field
- ○ Rubble field
- ○ Other:_________

How many trainers has the dog had, including the current one? ________________________

In addition to search training, the dog:

- ○ Is trained to promptly enter a crate/cage
- ○ Attended puppy class/basic education
- ○ Attended agility courses
- ○ Is trained for other work (e.g., hunting)
- ○ Is trained for shows
- ○ Was trained using a clicker
- ○ Attended obedience classes
- ○ Is used for pet therapy
- ○ Other: __________ ________

When the dog perceives that training is about to start, it appears: (describe with 3 terms)

1. ____
2. ____
3. ____

7

DOG TEMPERAMENT / PERSONALITY

Describe your dog using 3 adjectives:

1.____

2.____

3.____

Give a score from 0 (strongly disagree) to 5 (strongly agree):

Descriptor	0	1	2	3	4	5
Sensitive						
Fearful						
Playful						
Protective						
Jealous						
Sociable						
Calm						
Aggressive						
Territorial						
Affectionate						
Noisy						
Hyperactive						
Independent						
Shy						
Lazy						
Patient						
Energetic						
Exuberant						
Restless						
Obedient						
Trainable						
Intelligent						
Attentive						
Determined						
Docile						
Dominant						
Assertive						
Proud						
Opportunistic						
Gentle						
Nervous						
Cautious						
Submissive						

8

Figure S2. Word cloud displaying the most frequently mentioned descriptors, including only terms reported more than three times, with word size proportional to frequency.

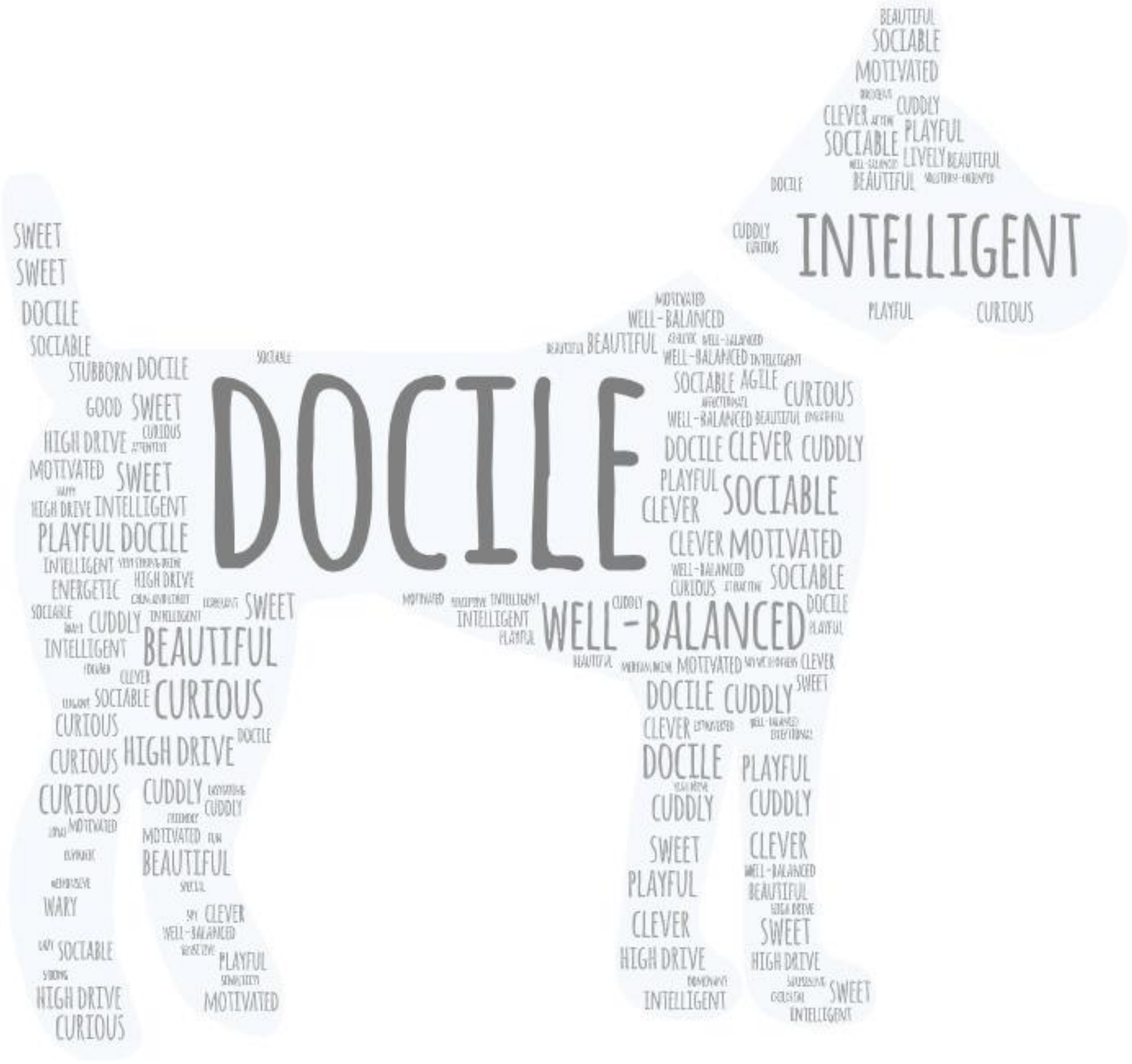

Supplement: Supplementary file 1 [file animals-16-00664-s001.zip › animals-4102701-supplementary.pdf]
